# Iron Chelators in the Management of Autoimmune-Induced Alopecia: A Focus on Hypoxia-Inducible Factor 1 Modulation and Hair Restoration

**DOI:** 10.3390/jcm14072133

**Published:** 2025-03-21

**Authors:** Andrea Pagani, Dominik Thor, Adriana C. Panayi, Silvan M. Klein, Sebastian Geis, Leonard Knoedler, Samuel Knoedler, Filippo A. G. Perozzo, Giuseppe Sofo, Rafael Loucas, Lukas Prantl, Dominik Duscher

**Affiliations:** 1 Department of Plastic, Reconstructive, and Aesthetic Surgery, University Hospital of Regensburg, Franz-Josef S. Allee 11, 93053 Regensburg, Germanysebastian.geis@ukr.de (S.G.); lknoedler@mgh.harvard.edu (L.K.); rafael.loucas@ukr.de (R.L.); lukas.prantl@ukr.de (L.P.); dominikduscher@me.com (D.D.); 2 Department of Research and Development, Geneva College of Longevity Science, GCLS, 1204 Geneva, Switzerland; d.thor@gcls.study; 3 Department of Oral and Maxillofacial Surgery, Charitè—Universitätsmedizin Berlin, 10117 Berlin, Germany; a.panayi@cantab.net; 4 Department of Plastic Surgery and Hand Surgery, Klinikum Rechts der Isar, Technical University of Munich, 80333 Munich, Germany; samknoe@gmail.com; 5 Department of Plastic and Reconstructive Surgery, Cleveland Clinic Foundation, Cleveland, OH 44195, USA; filippo.perozzo@gmail.com; 6 Instituto Ivo Pitanguy, Hospital Santa Casa de Misericordia Rio de Janeiro, Pontificia Universidade Catòlica do Rio de Janeiro, Rio de Janeiro 22453-900, Brazil; giuseppesofo93@gmail.com

**Keywords:** autoimmune-induced alopecia, iron chelators, HIF-1 alpha, hair restoration

## Abstract

Autoimmune-induced alopecia, such as alopecia areata, involves immune-mediated damage to hair follicles, leading to significant hair loss. Emerging therapies that stabilize hypoxia-inducible factor 1-alpha (HIF-1α) show promise in counteracting follicular degradation and supporting hair regrowth. This communication highlights the potential of iron chelators, specifically deferoxamine (DFO) and deferiprone (DFP), to stabilize HIF-1α by reducing iron availability, thereby promoting vascularization, cellular proliferation, and a regenerative environment in the hair follicle niche. Clinical trials with iron chelators demonstrated improvements in hair density, thickness, and elasticity, as well as a reduction in hair loss by up to 66.8% over six months. These findings underscore the therapeutic potential of iron chelators in autoimmune alopecia management. Future research should explore the synergistic use of iron chelators with immune-modulating therapies, positioning them as viable options in the evolving field of alopecia treatment.

## Autoimmune–Induced Alopecia, HIF–1 Modulation and Iron Chelators

Autoimmune-induced alopecia, which includes alopecia areata (AA) and cicatricial alopecia, is the condition in which the immune system erroneously targets hair follicles, leading to progressive hair loss [1]. Current therapeutic approaches, such as corticosteroids and immunosuppressive agents, provide limited efficacy and are often accompanied by lifelong undesirable side effects, particularly after long-term use. This therapeutic gap is driving the development of novel, targeted therapies aimed at modulating immune responses and promoting hair follicle resilience and regeneration. Among these emerging therapies, iron chelators have gained attention for their ability to stabilize hypoxia-inducible factor 1-alpha (HIF-1α), a crucial transcription factor in cellular repair and follicular regeneration [2,3]. Due to our interest and research focus on iron chelators in recent years, we have decided to share our perspective on the potential impact of iron-chelating agents in autoimmune alopecia.

Hair loss in autoimmune alopecia involves a multifaceted interplay of immune dysregulation, follicular miniaturization, and an impaired dermal microenvironment. Inflammation induced by autoimmune processes disrupts the hair growth cycle, prolonging the telogen (resting) phase and preventing re-entry into the anagen (active growth) phase [2]. HIF-1α, activated under hypoxic conditions, has been identified as a pivotal regulator of follicular function due to its role in upregulating genes involved in angiogenesis, cellular proliferation, and extracellular matrix production. However, in autoimmune alopecia, inflammation often leads to reduced oxygenation and nutrient supply to the dermal papilla—a critical structure supporting hair follicle health—thereby compromising HIF-1α activity and follicular vitality [1]. Beyond hypoxia, systemic iron homeostasis also influences follicular health. While low serum ferritin is linked to chronic telogen effluvium and alopecia areata, excess iron can drive oxidative stress via Fenton reactions, generating reactive oxygen species (ROS) that exacerbate follicular inflammation. Dysregulation of transferrin saturation and hepcidin levels may further contribute to immune dysfunction in hair loss disorders. Given their iron-sequestering properties, iron chelators could mitigate oxidative damage while modulating inflammatory responses in alopecia [4].

**Figure 1:**
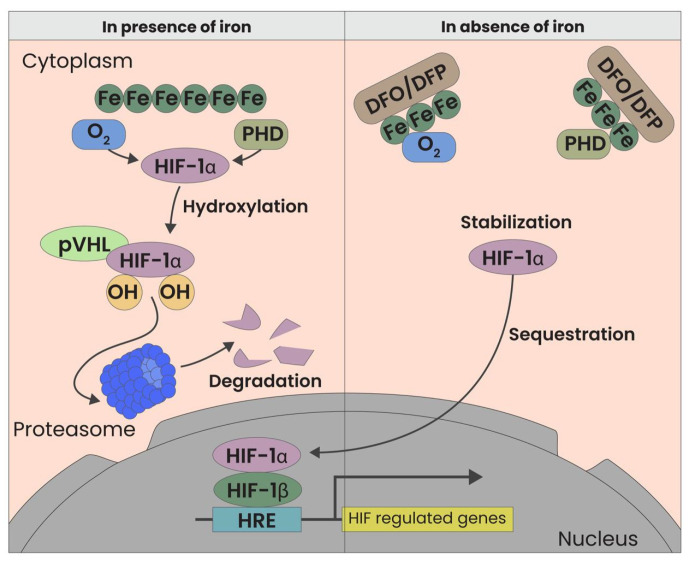
HIF Modulation through iron chelators. Both oxygen and the absence of iron are factors that influence HIF. In the presence of iron, HIF-1 alpha is rapidly degraded by pVHL. Conversely, in the absence of iron, HIF-1 alpha is stabilized, binds to the Beta subunit, and activates more than 100 downstream genes.

Iron chelators, such as Deferoxamine (DFO) and Deferiprone (DFP), offer a novel therapeutic approach by stabilizing HIF-1α levels. These agents inhibit prolyl-hydroxylase (PHD), an enzyme responsible for HIF-1α degradation under normoxic conditions [Figure 1]. We previously demonstrated that the reduction of local iron concentrations creates a hypoxia-mimetic environment that fosters dermal regeneration. This mechanism triggers a cascade of regenerative processes resembling those observed in wound healing, including enhanced neovascularization, collagen synthesis, and, primarily, fibroblast activity in aged fibroblasts [3]. In addition to these effects, HIF-1α stabilization may also modulate hair follicle stem cell (HFSC) populations, which are crucial for hair follicle cycling and sustained hair growth. HFSCs, residing in the bulge region of the follicle, are responsible for initiating the transition from telogen (resting phase) to anagen (growth phase). Recent studies suggest that hypoxic conditions enhance HFSC function by stabilizing HIF-1α, leading to the upregulation of growth factors such as VEGF and IGF-1, which are essential for stem cell proliferation, differentiation, and follicular regeneration [5,6]. By creating a hypoxia-mimetic environment, iron chelators could indirectly support HFSC survival and activation, thereby improving follicular regeneration and hair quality. Further research is needed to determine whether the regenerative effects of deferoxamine and deferiprone directly influence HFSCs or are mediated through paracrine signaling from fibroblasts and endothelial cells. Such effects could also regenerate the damage caused by autoimmune inflammation and, by proxy, restore a favorable microenvironment for hair follicle growth.

Recent clinical trials have begun to substantiate the therapeutic potential of iron chelators in hair and skin rejuvenation. Notably, a blinded study conducted by our group demonstrated significant clinical improvements in patients with androgenic alopecia (AGA). Over a nine-month period, participants experienced measurable benefits, including a 66.8% reduction in hair shedding after six months and increases in hair density (+14.3%), thickness (+7.2%), and shine and elasticity (+20.3%). These findings highlight the efficacy of HIF-1α modulation in improving hair quality and reducing hair loss [7].

Iron chelators offer multiple advantages over traditional therapies. Beyond stabilizing HIF-1α, they mitigate oxidative stress by binding iron ions, thereby reducing the formation of reactive oxygen species (ROS) that exacerbate follicular inflammation and degeneration. Iron chelators may also exert immunomodulatory effects by influencing macrophage polarization and cytokine expression. By reducing intracellular iron, they can shift macrophages toward an anti-inflammatory M2 phenotype and suppress NF–κB signaling, thereby decreasing levels of TNF-α and IL-15, key cytokines involved in autoimmune follicular destruction. This suggests that iron chelation may not only promote regeneration but also attenuate immune-mediated damage in alopecia.

This dual mechanism positions iron chelators as a potential approach to address both the inflammatory and degenerative aspects of autoimmune alopecia. Furthermore, the safety and tolerability of iron chelation therapy, as evidenced by our most recent clinical trial, present significant advantages [7]. Unlike systemic immunosuppressants or corticosteroids, topical application of iron chelators minimizes systemic exposure and delivers direct benefits to affected hair follicles, reducing the risk of adverse effects.

The promising results of iron chelation therapy suggest potential applicability for autoimmune-induced alopecia, particularly when combined with other treatments. For instance, integrating iron chelators with Janus kinase (JAK) inhibitors, known for their immunomodulatory effects, may provide synergistic benefits in conditions like alopecia areata [8]. Recent studies have highlighted the effectiveness of selective JAK3 inhibitors such as Ritlecitinib, which demonstrated significant hair regrowth in patients with severe AA. Clinical trials have reported that up to 50% of patients achieved a SALT30 score (scalp hair loss ≤ 30%) after 24 weeks of treatment, along with improvements in eyelash and eyebrow growth. In another manuscript of King et al. [9], it was reported that promising results have led to the FDA approval of Baricitinib, a selective JAK1/JAK2 inhibitor, for the treatment of severe alopecia areata. This medication has demonstrated significant efficacy in clinical trials, showing substantial hair regrowth not only on the scalp but also on eyelashes and eyebrows. Baricitinib’s approval marks a significant milestone, providing an effective option for patients with refractory AA [8].

Additionally, autoimmune alopecia may be exacerbated by systemic inflammatory conditions, as evidenced by cases reported by Krivda et al. [10] of extensive alopecia areata following drug-induced hypersensitivity syndrome (DIHS/DRESS). This underscores the importance of considering systemic inflammatory triggers when developing treatment protocols. The potential interplay between immune dysregulation, follicular damage, and systemic inflammation suggests that stabilizing HIF-1α in combination with immunomodulatory agents may offer a comprehensive therapeutic strategy.

Beyond alopecia, iron chelators have potential applications in other dermatologic conditions involving hypoxic stress or chronic inflammation. Conditions such as psoriasis, rosacea, and even chronic wounds may benefit from the regenerative effects of HIF-1α stabilization [11,12]. Future research should focus on large-scale, randomized controlled trials to validate the long-term efficacy and safety of iron chelation therapy across diverse patient populations. An important consideration for the clinical application of iron chelation therapy is its potential for personalized treatment approaches. Given the variability in autoimmune alopecia presentation and progression, identifying biomarkers and patient-specific factors could help optimize therapeutic outcomes. Genetic predisposition, systemic iron levels, and inflammatory markers may play a role in determining responsiveness to iron chelators. For instance, variations in genes regulating iron metabolism, such as HFE or FPN–1, have been implicated in differential iron homeostasis and could influence patient responses to chelation therapy [13]. Additionally, patients with elevated oxidative stress or hypoxia-related dysregulation—measured via serum ferritin, transferrin saturation, or HIF-1α expression levels—might benefit more from iron chelation strategies. Disease severity may also help tailor treatment protocols by identifying those who require combination therapies, such as iron chelators with JAK inhibitors [14,15].

Novel research exploring molecular and genetic predictors of response could facilitate personalized treatment strategies, maximizing benefits for individual patients. Investigating the downstream signaling pathways of HIF-1α activation and identifying biomarkers associated with therapeutic response may further refine and expand the clinical utility of this innovative approach.

While direct studies on JAK inhibitors and iron chelators in alopecia are lacking, oxidative stress is known to enhance JAK–STAT signaling in autoimmune diseases. Since iron chelators reduce ROS and inflammation, their combination with JAK inhibitors could enhance therapeutic efficacy. Investigating the interplay between iron metabolism, immune modulation, and JAK–STAT pathway activation may provide new insights for optimizing treatment strategies in severe alopecia. These approaches, targeting both immune modulation and iron homeostasis, offer promising avenues for enhancing hair regrowth and improving patient outcomes. With the growing body of evidence supporting their efficacy, future research should prioritize optimizing treatment regimens and exploring combination therapies to achieve maximal therapeutic benefit in patients suffering from autoimmune alopecia.

## References

[1] Ahn D., Kim H., Lee B., Hahm D.H. (2023). Psychological Stress-Induced Pathogenesis of Alopecia Areata: Autoimmune and Apoptotic Pathways. Int. J. Mol. Sci..

[2] Pratt C.H., King L.E., Messenger A.G., Christiano A.M., Sundberg J.P. (2017). Alopecia areata. Nat. Rev. Dis. Primers.

[3] Pagani A., Kirsch B.M., Hopfner U., Aitzetmueller M.M., Brett E.A., Thor D., Mela P., Machens H.G., Duscher D. (2021). Deferiprone Stimulates Aged Dermal Fibroblasts via HIF-1α Modulation. Aesthet. Surg. J..

[4] Garrido-Rodríguez V., Álvarez-Ríos A.I., Olivas-Martínez I., Pozo-Balado M.d.M., Bulnes-Ramos Á., Leal M., Pacheco Y.M. (2022). Dysregulation of iron metabolism modulators in virologically suppressed HIV-infected patients. Front. Immunol..

[5] Blanpain C., Fuchs E. (2006). Epidermal Stem Cells of the Skin. Annu. Rev. Cell Dev. Biol..

[6] Lopez-Lazaro M. (2008). The Warburg Effect: Why and How Do Cancer Cells Activate Glycolysis in the Presence of Oxygen?. Anti-Cancer Agents Med. Chem.-Anti-Cancer Agents.

[7] Thor D., Pagani A., Bukowiecki J., Houschyar K.S., Kølle S.-F.T., Wyles S.P., Duscher D. (2023). A Novel Hair Restoration Technology Counteracts Androgenic Hair Loss and Promotes Hair Growth in A Blinded Clinical Trial. J. Clin. Med..

[8] Ramírez-Marín H.A., Tosti A. (2022). Evaluating the Therapeutic Potential of Ritlecitinib for the Treatment of Alopecia Areata. Drug Des. Devel. Ther..

[9] King B., King B., Ohyama M., Ohyama M., Kwon O., Kwon O., Zlotogorski A., Zlotogorski A., Ko J., Ko J. (2022). Two Phase 3 Trials of Baricitinib for Alopecia Areata. N. Engl. J. Med..

[10] Krivda L.K., Campagna L.J., Mignano M.S., Cho C.S. (2022). Prolonged Drug-Induced Hypersensitivity Syndrome/DRESS with Alopecia Areata and Autoimmune Thyroiditis. Fed. Pract..

[11] Boehncke W.H., Schön M.P. (2015). Psoriasis. Lancet.

[12] Van Zuuren E.J., Arents B.W.M., Van Der Linden M.M.D., Vermeulen S., Fedorowicz Z., Tan J. (2021). Rosacea: New Concepts in Classification and Treatment. Am. J. Clin. Dermatol..

[13] Ganz T., Nemeth E. (2012). Hepcidin and iron homeostasis. Biochim. Biophys. Acta.

[14] Hu X., Li J., Fu M., Zhao X., Wang W. (2021). The JAK/STAT signaling pathway: From bench to clinic. Sig. Transduct. Target Ther..

[15] Liu M., Gao Y., Yuan Y., Yang K., Shen C., Wang J., Tian J. (2023). Janus Kinase Inhibitors for Alopecia Areata: A Systematic Review and Meta-Analysis. JAMA Netw. Open.

